# Virtual screening, drug-likeness analysis, and molecular docking study of potential severe acute respiratory syndrome coronavirus 2 main protease inhibitors

**DOI:** 10.3906/kim-2103-20

**Published:** 2021-09-16

**Authors:** Nikola V. NEDELJKOVIĆ, Miloš V. NIKOLIĆ, Ana S. STANKOVIĆ, Nevena S. JEREMIĆ, Dušan Lj. TOMOVIĆ, Andriana M. BUKONJIĆ, Gordana P. RADIĆ, Marina Ž. MIJAJLOVIĆ

**Affiliations:** Department of Pharmacy, Faculty of Medical Sciences, University of Kragujevac, Kragujevac, Serbia

**Keywords:** SARS-CoV-2 main protease, virtual screening, AutoDock Vina, molecular dynamics, MM/GBSA

## Abstract

Due to the length of time required to develop specific antiviral agents, the World Health Organization adopted the strategy of repurposing existing medications to treat Coronavirus disease 2019 infection. Severe acute respiratory syndrome coronavirus 2 (SARS-CoV-2) main protease is possible biological target for potential antiviral drugs. We selected various compounds from PubChem database based on the structure of main protease inhibitors in Protein Data Bank database. Ten compounds showed nontumorigenic and nonmutagenic potential and met Egan’s and Lipinski’s rules. Molecular docking analysis was performed using AutoDock Vina software. Based on number and type of key binding interactions, as well as docking scores, we selected compounds **6**, **8**, and **17** that demonstrated the highest binding affinity for the target protein. Molecular dynamics simulations were then carried out on the protein-top docked ligand complexes which were subjected to molecular mechanics/generalized Born and surface area calculations. The molecular dynamics simulation results indicated that protein-top docked ligand complexes showed good conformational stability. Among analyzed molecules, compound **17** emerged as the best in silico hit based on the docking score, MM/GBSA binding energy and MD results.

## 1. Introduction

Integration between computational and experimental strategies has a great value in the identification and developmentof novel promising compounds. Among a many principles of drug discovery, where high-cost rates are a major problem, computer-aided drug design (CADD) methodologies are time-saving and cost-effective alternatives [1]. Molecular docking analysis can identify promising compounds that might represent future solutions in critical areas of human health [2].

In December 2019, a novel coronavirus was discovered in Wuhan, a city in China’s Hubei Province [3]. The virus has spread rapidly to more than 200 countries in the world, after which World Health Organization (WHO) declared a global pandemic, named the virus severe acute respiratory syndrome coronavirus 2 (SARS-CoV-2) and the viral disease as coronavirus disease 2019 (COVID-19) [4]. On March 14, 2021, there were 119,605,581 coronavirus cases and more than two million fatal outcomes^1^.

COVID-19 infection is transmitted through large droplets which were generated during coughing and sneezing and may cause disorder ranging from asymptomatic to fatal disease [5]. SARS-CoV-2 infects the lower respiratory tract with potential to cause fatal pneumonia in elderly patients. Furthermore, infection can progress to hypoxemia, difficulty in breathing and acute respiratory distress syndrome (ARDS) [6].

Some of SARS-CoV-2 therapeutic drug design targets include envelop proteins, membrane proteins, proteases, nucleocapsid protein, hemagglutinin esterase, and helicase [7]. The chymotrypsin-like cysteine protease (3CLPro), also known as main protease of the SARS-CoV-2 (SARS-CoV-2 Mpro), cleaves the viral polypeptides to generate various nonstructural proteins critical for viral replication [8,9].

**Figure 1:**
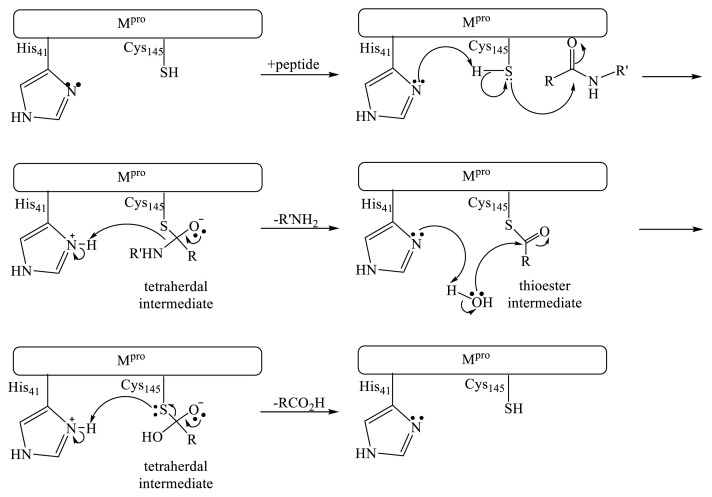
Proteolysis mechanism of the SARS-Cov-2 main protease.

Mpro belongs to the enzymes class called cysteine proteases. These proteases usually contain cysteine and histidine residues in the catalytic active site, which catalyze the cleavage of polypeptides by the following mechanism (Figure 1) [10]. There are numerous reports of reversible cysteine protease inhibitors including aldehydes [11], cyclic ketones [12], amidomethyl ketones [13], nitriles [14], and 1,2-dicarbonyl compounds [15]. The carbonyl group of these compounds reversibly reacts with cysteine sulfur atom in active site forming a covalent bond [16].

**Figure 2:**
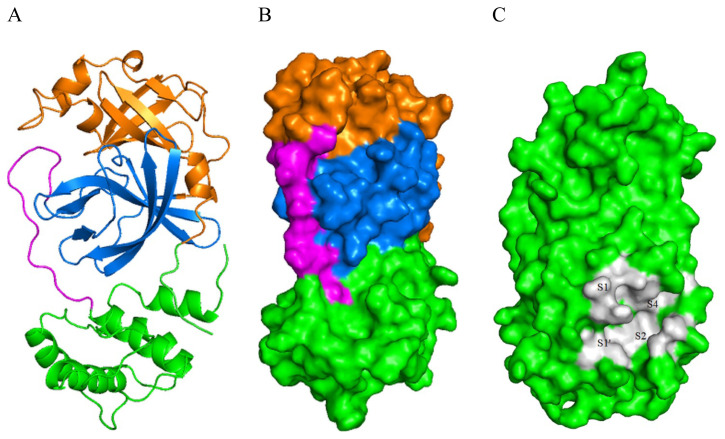
A) Cartoon presentation of the SARS-CoV-2 Mpro protein backbone highlighting the constituent domains. Domain I is colored brown, domain II blue, domain III green and the loop connecting domains II and III is colored pink. B) Surface depiction of the enzyme with domains. C) A close up view of the active sites showing its four subpockets.

SARS-Cov-2 Mpro consists of three domains: domain I (residues 8–101), domain II (residues 102–184), and domain III (residues 201–303). Domain II and III connecting region is marked as a long loop (residues 185–200) (Figure 2). The active site of the enzyme is located between domains I and II. It is divided into four subsites named S1, S1′, S2, and S4 [17].

At this point, on the world market, remdesivir is the only applicable drug for the COVID-19 infection treatment approved by Food and Drug Administration (FDA) [18]. Repurposing of available medications has been the standard care in the treatment of SARS-CoV-2 patients. These old drugs include antiviral agents such as remdesivir [19], favipiravir [20], ribavirin [21], lopinavir-ritonavir [22], and oseltamivir [23], azithromycin as an antibacterial agent [24], chloroquine and hydroxychloroquine as antimalarial agents [25], tocilizumab [26] and interferons [27,28] as immunomodulatory agents, glucocorticoids such as methylprednisolone and dexamethasone [29,30] as well as traditional Chinese medicines [31]. Although all these drugs show some potential in the treatment of COVID-19 infection, evidence from living systematic reviews and network metaanalyses suggest that glucocorticoids probably reduce mortality and mechanical ventilation in patients with severe COVID-19 infection, while remdesivir probably reduces length of hospital stay [32]. The effects of current therapeutic options are very uncertain because most of the conducted clinical trials were small and had important limitations. The computational methods of drug repurposing have become an attractive and rapid strategy to identify known drugs that can efficiently treat COVID-19 infection. Although there is a lack of evidence of their clinical efficacy, based on these in silico methods there is a good chance to select one of the approved drugs which could eradicate SARS-CoV. Drugs repurposed by computational methods that target main protease (Mpro), papain-like protease (PLpro), spike protein (Spro), helicase, RNA-dependent RNA polymerase (RdRp) and type 2 transmembrane serine protease (TMPRSS2) include above all: antiviral drugs [33], anticoagulants, itraconazole [34], ergotamine, dihydroergotamine, bromocriptine [35], various peptide-based drugs from DrugBank [36], and organosulfur compounds [37].

On the other hand, according to the WHO, more than 100 vaccine candidates at this moment are under development^2^, many of which are already in the human trial phase [38,39]. Despite the great potential of vaccine use in the future, there could be significant limitations concerning poor public trust or safety. There is a need for a safe and effective treatment of COVID-19 infection in order to save severely ill patients.

Molecular docking study is based on the hypothesis that the selected compounds are capable of interfering with the active site of the SARS-CoV-2 main protease and cause inhibition of its activity. Molecular dynamics (MD) simulations were carried out on the protein-top docked ligand complexes to get a better understanding of the compounds affinity for SARS-CoV-2 Mpro active site. Protein-ligand complexes were then subjected to molecular mechanics/generalized Born and surface area (MM/GBSA) calculations to estimate the corresponding average binding free energies. By comparing the inhibition profiles of selected compounds and cocrystallized ligands for SARS-CoV-2 main protease, we can estimate their potential as antiviral agents in the treatment of COVID-19 infection.

## 2. Materials and methods

### 2.1. Hardware

Molecular docking studies were carried out on the Lenovo Intel® Core (TM) i5-10210U CPU, processor @1.60 GHz 2.11 GHz, memory (RAM) 8.00 GB, 64-bit Operating system, Windows 10 Pro. MD simulations and MM/GBSA calculations were carried out on Intel Core i9-9900K CPU 3.60 GHz, memory (RAM) 16 GB, Graphics NVIDIA GeForce RTX 2070 SUPER, Operating system Ubuntu 20.04.2 LTS 64-bit.

### 2.2. Virtual screening

**Figure 3:**
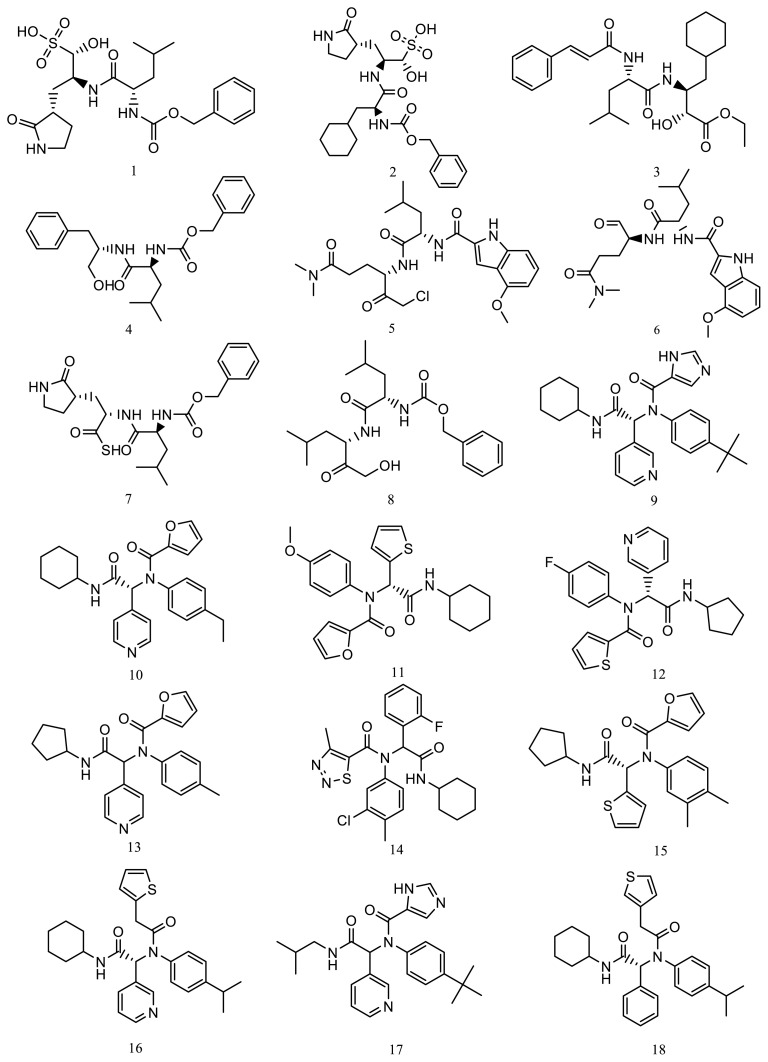
The structures of the initial compounds.

Based on the protein structure of the SARS-CoV-2 main protease (Enzyme Commission (EC) number: 3.4.22.69), a search of available ligands was performed in the Protein Data Bank (PDB) database^3^. The set of ligand molecules studied in this work included compounds with PDB ligand ID X77 (PubChem Compound ID: 145998279), ID V2M (PubChem Compound ID: 11561899), ID K36 (PubChem Compound ID: 118737648) and its three-dimensional structurally similar bioactive compounds obtained from PubChem database [40]. There were 10 compounds similar to X77, 4 compounds similar to V2M, and 4 compounds similar to K36, which formed the initial set of 18 compounds **(1–18)** (Figure 3).

### 2.3. Physicochemical and toxicological properties and drug-likeness calculations

Physicochemical descriptors, lipophilicity, and water solubility of the studied compounds were analyzed using SwissADME web-based interface [41]. The compounds were checked for drug-likeness by using Lipinski’s rule of five and Egan’s rule, obtaining the molecular properties and bioactivity prediction from Molinspiration^4^. The Lipinski’s rule was examined by the following attributes: hydrogen bond donors (not more than 5), hydrogen bond acceptors (not more than 10), partition coefficient (not more than 5), and molecular weight (less than 500 g/mol) [42]. The Egan’s rule was examined by partition coefficient (not more than 5.88) and total polar surface area (not more than 131.6 Å) [43]. Toxicological properties of the tested compounds were calculated using OSIRIS Data Warrior [44]. The simplified molecular-input line-entry system (SMILES) format of the selected compounds was obtained from PubChem database.

### 2.4. Ligand preparation

All the selected molecules were drawn using 2D option of ChemDraw Ultra 7.0 and saved in cdx format. Thereafter, the designed molecules were individually optimized using AM1 semiempirical quantum chemical methods in Chem3D Ultra 7.0 platform and saved in pdb format [45]. Furthermore, these molecules were imported into the Mercury 3.10.2 [46] and converted into the mol2 format. In order to prepare selected compounds for the docking calculations, the Graphical User Interface program AutoDockTools 1.5.6. [47] was used to add Gasteiger changes, set rotatable bonds, and save selected molecules in pdbqt format.

### 2.5. Selection and preparation of receptors

The X-ray crystal structures of the SARS-CoV-2 main protease were retrieved from the Protein data bank having PDB IDs 6W63, 6XHM [48], and 7D1M [4], respectively (Table 1).

Receptor data were opened using BIOVIA Discovery Studio Visualizer 17.2.0.16349^5^in order to remove the cocrystallized ligands, water molecules, and unnecessary receptor chains. All docking calculations were performed on chain A. Before the docking process, AutoDockTools 1.5.6. was used to prepare the protein data for AutoDock Vina by assigning hydrogens and converting protein structures from the pdb to pdbqt format.

### 2.6. Docking methodology

In order to carry out the docking calculations, we used the AutoDock Vina software [49] with the default scoring function. The quality of results obtained from AutoDock Vina software is comparable to those from AutoDock software [50]. In this docking simulation, we used semiflexible docking protocols in which the target protein was kept rigid. Based on the cocrystal X77 location coordinates in the 6W63 crystal structure which were set at x = −19.34, y = 18.376, and z = −27.228, a grid box of 42, 28, and 32 points in x-, y-, and z-direction, respectively, with grid spacing of 0.375 Å were built and centered on the cocrystal ligand. The location coordinates of native V2M ligand in the 6XHM crystal structure were set at x = 10.214, y = 15.528, and z = 27.34, and based on that, a grid box of 30, 38, and 30 points was built and centered on the cocrystal ligand. Finally, the coordinates of the cocrystal K36 ligand in the 7D1M crystal structure were x = 9.585, y = 6.174, and z = 27.224, while grid box size was set at x = 30, y = 26, and z = 32. Binding site similarity analysis between tested and cocrystal ligands were performed in order to estimate the antiviral potential of the selected compounds. Maximum of nine poses were generated for the each tested compound. The final visualization of the docked structure was performed using BIOVIA Discovery Studio Visualizer.

### 2.7. Validation of docking methodology

For validation of the docking protocol, the cocrystal ligands were extracted and redocked into the active sites of the target protein. Root-mean-square deviation (RMSD) value between the docked structure and native inhibitor conformation in each complex was calculated. RMSD value cut-off less than 2 Å is considered a good prediction for computed ligand-protein conformation.

### 2.8. Molecular dynamics simulations and MM/GBSA calculations

MD simulations for interaction analysis were performed using Schrödinger package. We carried out MD analysis using Desmond 2020-4 version^6^ for the free proteins, proteins cocrystallized with inhibitors, and protein-best docked ligand complexes after molecular docking study. SARS-CoV-2 Mpro-docked ligand complexes were placed in the orthorhombic box with a distance of 10 Å to create a hydration model using TIP3P water model [51]. Free proteins, proteins cocrystallized with inhibitors, and protein-ligand complexes were neutralized with 0.15M NaCl. MD simulations run of 20 ns was set up at a constant temperature (300K) and constant pressure (1.01325 bar) with recording intervals of 1.2 ps for energy and 4.8 ps for trajectory. We performed MD simulations under the NPT ensemble using OPLS3e force field [52]. The cut-off radius for van der Waals and electrostatic interactions were set to 9 Å. Interaction fractions of residues involved in protein-ligand contacts during MD simulations were interpreted using the Simulation Interaction Diagram tool in Maestro^7^. The simulation trajectories of SARS-CoV-2 Mpro alone and its complexes with cocrystallized inhibitors and ligands were analyzed for the outcomes of RMSD and root mean square fluctuation (RMSF).

The binding energies between the SARS-CoV-2 Mpro and top three docked ligands (**6**, **8**, and **17**) were computed using the MM/GBSA method [53]. The parameter average binding free energy (ΔG bind) with standard deviation was calculated using the thermal_mmgbsa.py script [54]. MM/GBSA binding energies were calculated using frames from the 10 ns of each system trajectory.

## 3. Results

### 3.1. Physicochemical and toxicological properties and drug-likeness

In this work, in silico study of the tested compounds was performed to predict physicochemical properties, lipophilicity, water solubility, toxicological properties, and drug-likeness. The results are summarized in Tables 2 and 3. Molecular properties of the initial compounds were evaluated using Molinspiration to fit into Lipinski’s rule of five and Egan’s rule. Ten of the 18 compounds met the necessary criteria to be included in further research. These 10 compounds (**3**, **4**, **6**, **8**, **9**, **10**, **11**, **12**, **13**, and **17**) did not show violations for Lipinski’s and Egan’s rules and could be considered completely safe in terms of their mutagenic and tumorigenic potential.

### 3.2. Validation of molecular docking

For evaluation of the accuracy of molecular docking protocol, the cocrystal ligand has to be redocked into the active site. RMSD value was calculated for all three cocrystal ligands by superimpose native and redocked conformation on each other using BIOVIA Discovery Studio Visualizer. The redocked conformations of the cocrystal ligands are highlighted with green color in Table 4. Since the calculated RMSD values for all three cocrystal ligands were less than 2 Å, we concluded that performed molecular docking protocol could be considered valid.

### 3.3. Molecular docking analysis

In this in silico study various compounds obtained from PubChem database were docked into the active site of SARS-CoV-2 main protease. Within the molecular docking analysis, the interactions of specific amino acid residues that participate in drug-protein interactions were examined and corresponding docking scores were calculated. The key binding interactions are considered to be those interactions that the cocrystal achieves in the active site of the target proteins. The docking scores for nine different conformations were generated for each investigated molecule. Tested compounds were divided into two groups according to different binding affinity. Binding affinity analysis was performed based on the following criteria: number and type of key binding interactions with special reference to hydrogen bonds, as well as docking score of the best binding ligand conformation. The first group consisted of selected compounds which were bound to the target protein with decreased affinity (Table 5), while the second group included tested compounds that demonstrated higher binding affinity for the target enzyme (Table 6).

Binding mode analysis of the tested compounds into the active site of the SARS-CoV-2 main protease revealed that compounds **3**, **5**, **9**, **10**, **11**, **12**, and **13** bound to the target enzyme with decreased affinity. Among these molecules, compound **9** showed the best binding affinity towards 6W63 and 7D1M. The values of free binding energies in this group of compounds were in the range of −6.6 kcal/mol to −8.1 kcal/mol. Table 5 shows the key binding interactions and docking scores of compounds with lower binding affinity for the SARS-CoV-2 main protease. Table 6 summarizes the main binding parameters of molecules with higher affinity for the target enzyme. Based on the binding affinity analysis described above, it can be concluded that molecules **6**, **8**, and **17** showed the greatest potential for all three target proteins.

The highest value of the free binding energy (−8.1 kcal/mol) and the largest number of significant interactions was achieved by compound **17** towards 6W63.

### 3.4. Visualization of molecular docking results

**Figure 4:**
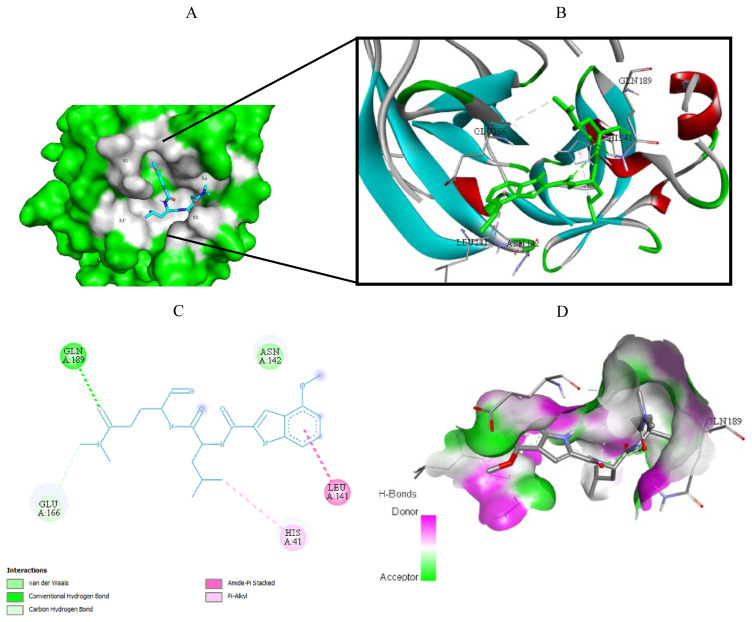
Compound **6** docked into the active site of 6W63. A) Best ligand conformation in the binding pocket of protein. B) and C) 2D and 3D summary views of all interactions achieved by compound **6** into the active site of 6W63 (hydrogen bonds were presented as green dash lines). D) 3D visualization of hydrogen bond donors and acceptors distribution of compound.

**Figure 5:**
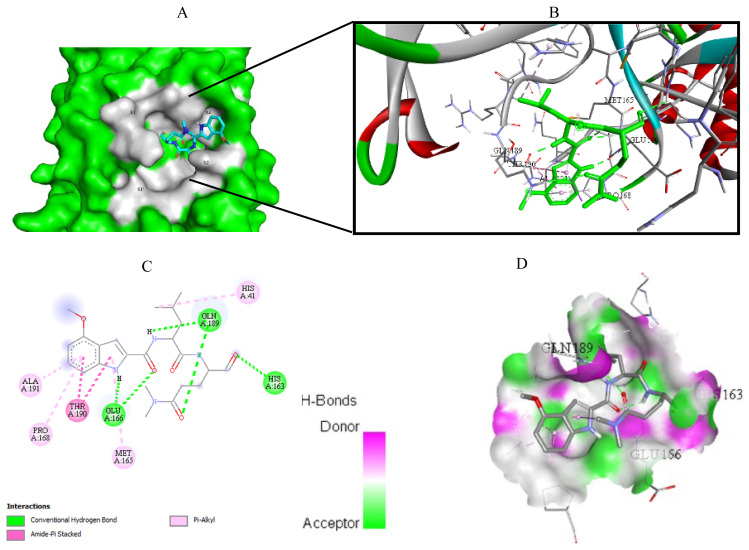
Compound **6** docked into the active site of 6XHM. A) Best ligand conformation in the binding pocket of protein. B) and C) 2D and 3D summary views of all interactions achieved by compound **6** into the active site of 6XHM (hydrogen bonds were presented as green dash lines). D) 3D visualization of hydrogen bond donors and acceptors distribution of compound.

**Figure 6:**
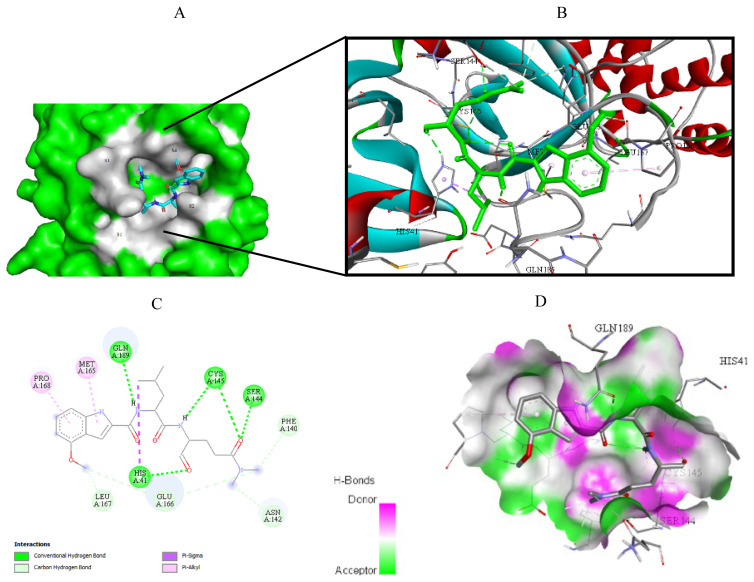
Compound **6** docked into the active site of 7D1M. A) Best ligand conformation in the binding pocket of protein. B) and C) 2D and 3D summary views of all interactions achieved by compound **6** into the active site of 7D1M (hydrogen bonds were presented as green dash lines). D) 3D visualization of hydrogen bond donors and acceptors distribution of compound.

**Figure 7:**
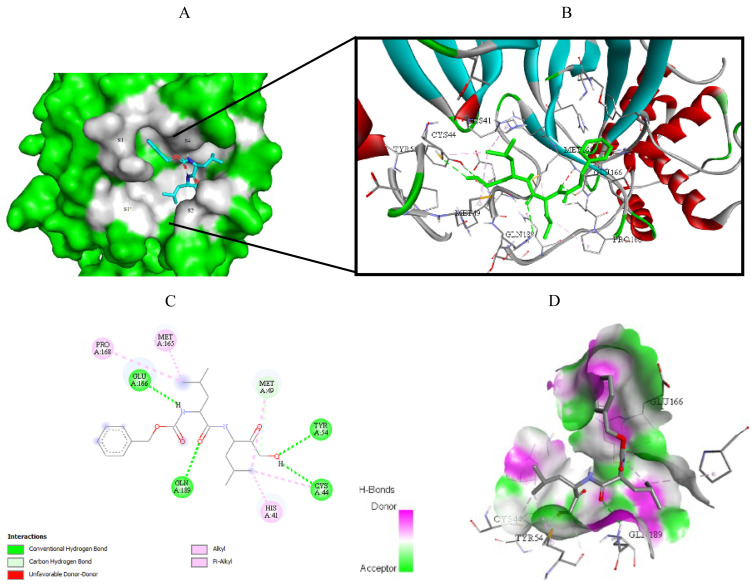
Compound **8** docked into the active site of 6W63. A) Best ligand conformation in the binding pocket of protein. B) and C) 2D and 3D summary views of all interactions achieved by compound **8** into the active site of 6W63 (hydrogen bonds were presented as green dash lines). D) 3D visualization of hydrogen bond donors and acceptors distribution of compound.

**Figure 8:**
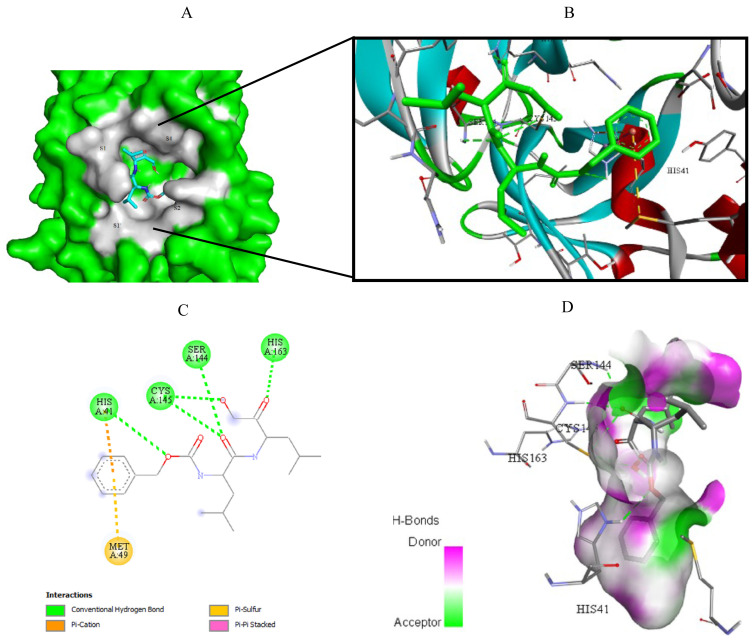
Compound **8** docked into the active site of 6XHM. A) Best ligand conformation in the binding pocket of protein. B) and C) 2D and 3D summary views of all interactions achieved by compound **8** into the active site of 6XHM (hydrogen bonds were presented as green dash lines). D) 3D visualization of hydrogen bond donors and acceptors distribution of compound.

**Figure 9:**
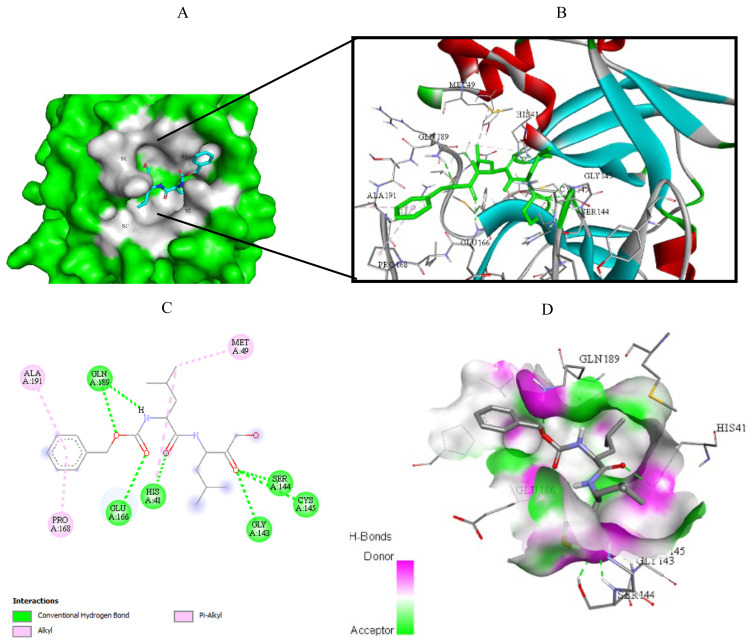
Compound **8** docked into the active site of 7D1M. A) Best ligand conformation in the binding pocket of protein. B) and C) 2D and 3D summary view of all interactions achieved by compound **8** into the active site of 7D1M (hydrogen bonds were presented as green dash lines). D) 3D visualization of hydrogen bond donors and acceptors distribution of compound.

**Figure 10:**
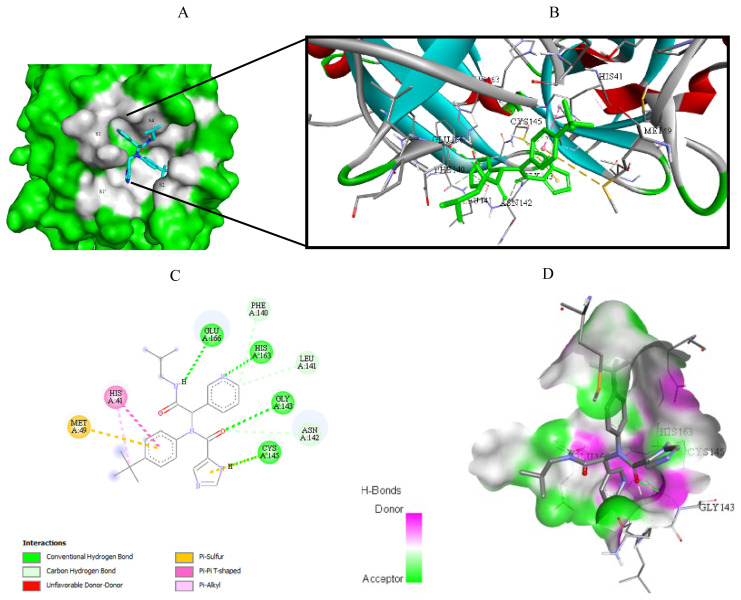
Compound **17** docked into the active site of 6W63. A) Best ligand conformation in the binding pocket of protein. B) and C) 2D and 3D summary views of all interactions achieved by compound **17** into the active site of 6W63 (hydrogen bonds were presented as green dash lines). D) 3D visualization of hydrogen bond donors and acceptors distribution of compound.

**Figure 11:**
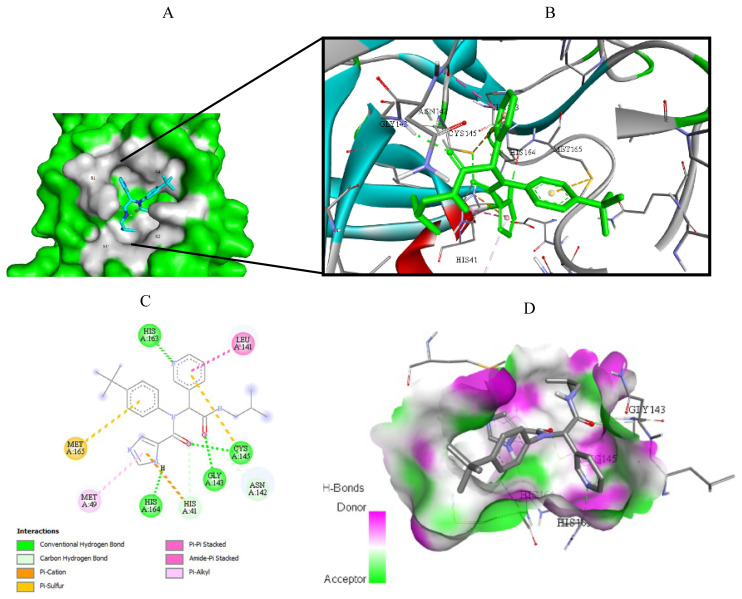
Compound **17** docked into the active site of 6XHM. A) Best ligand conformation in the binding pocket of protein. B) and C) 2D and 3D summary views of all interactions achieved by compound **17** into the active site of 6XHM (hydrogen bonds were presented as green dash lines). D) 3D visualization of hydrogen bond donors and acceptors distribution of compound.

**Figure 12:**
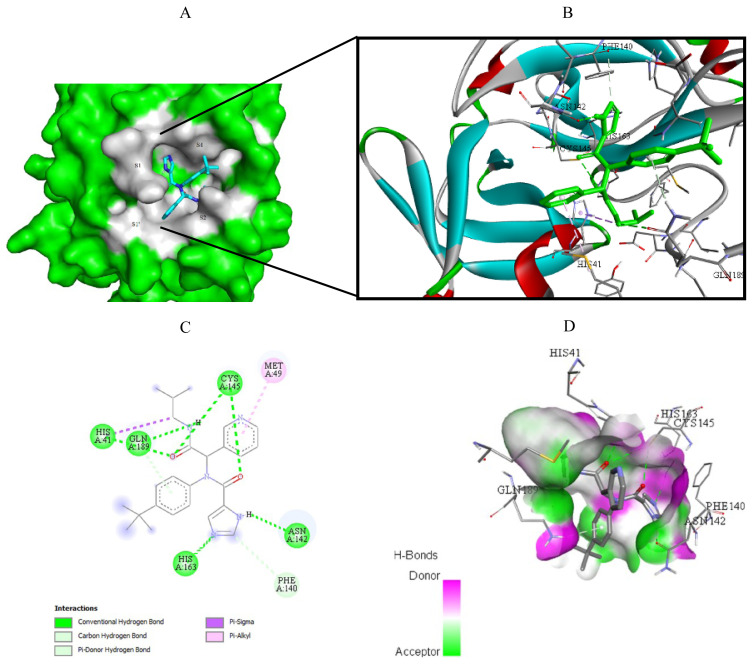
Compound **17** docked into the active site of 7D1M. A) Best ligand conformation in the binding pocket of protein. B) and C) 2D and 3D summary views of all interactions achieved by compound **17** into the active site of 7D1M (hydrogen bonds were presented as green dash lines). D) 3D visualization of hydrogen bond donors and acceptors distribution of compound.

Visualization of the molecular docking results was performed using BIOVIA Discovery Studio Visualizer and Pymol 2.4.1^8^. The lowest energy conformations of the ligands were docked into the active site of target protein. Docking visualization was presented through 2D and 3D view of the key binding interactions. 3D visualization of hydrogen bond interactions was also presented. In order to achieve visibility of the docked ligand into the protein structure, ligands were shown as sticks (blue) in the binding pocket of the protein (green) (Figures 4–12).

### 3.5. Molecular dynamics simulations

**Figure 13:**
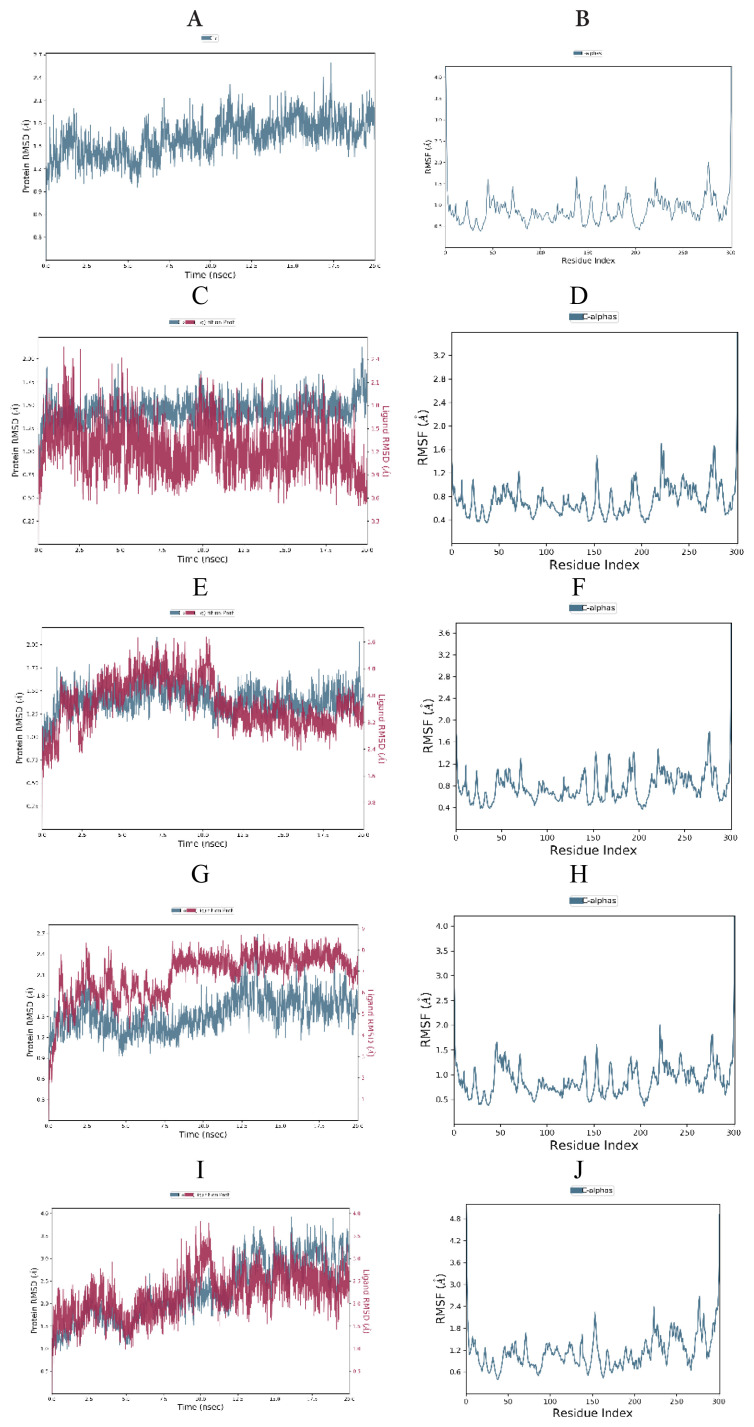
RMSD and RMSF analysis of MD simulation trajectory obtained for: (A,B) free protein (6XHM), (C,D) protein-cocrystallized ligand complex, (E,F) protein-compound **6** complex, (G,H) protein-compound **8** complex, (I,J) protein-compound **17** complex.

**Figure 14:**
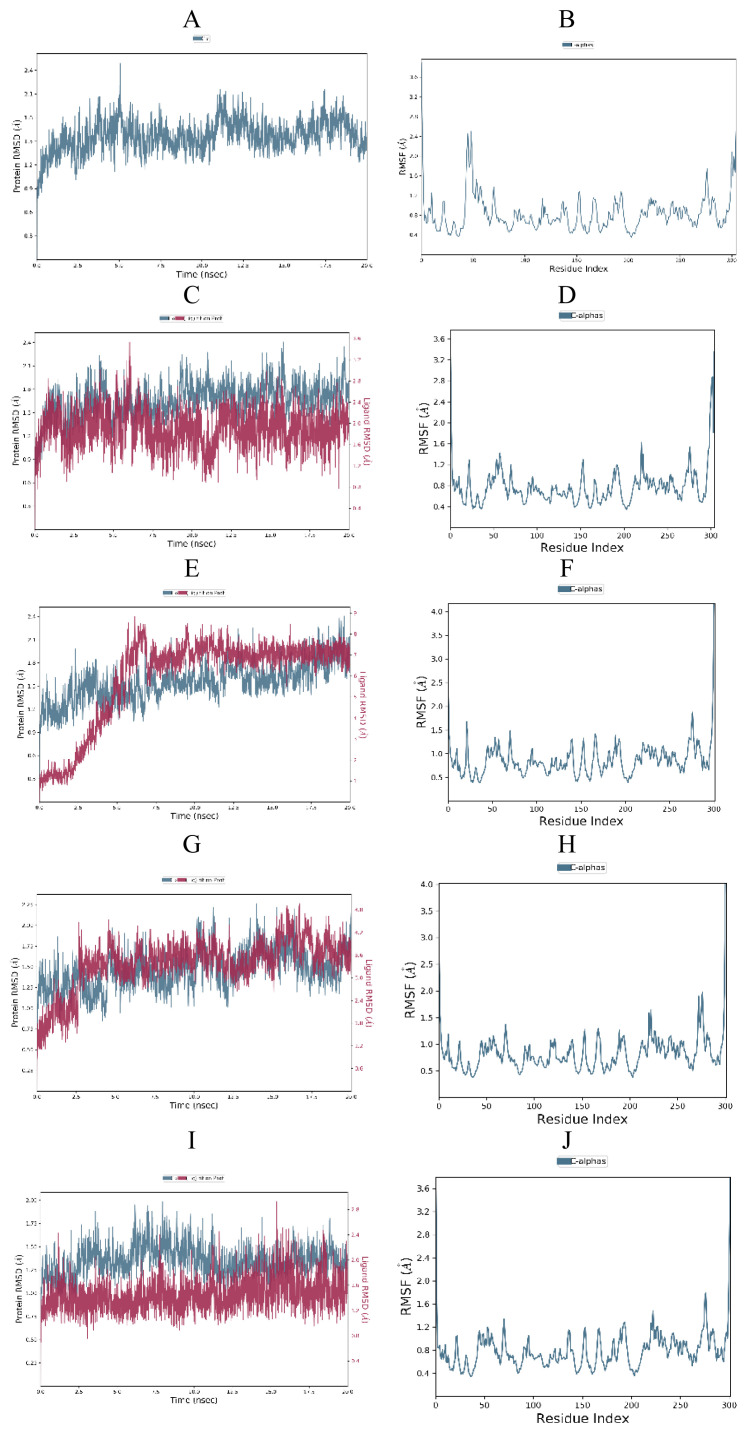
RMSD and RMSF analysis of MD simulation trajectory obtained for: (A,B) free protein (7D1M), (C,D) protein-cocrystallized ligand complex, (E,F) protein-compound **6** complex, (G,H) protein-compound **8** complex, (I,J) protein-compound **17** complex.

**Figure 15:**
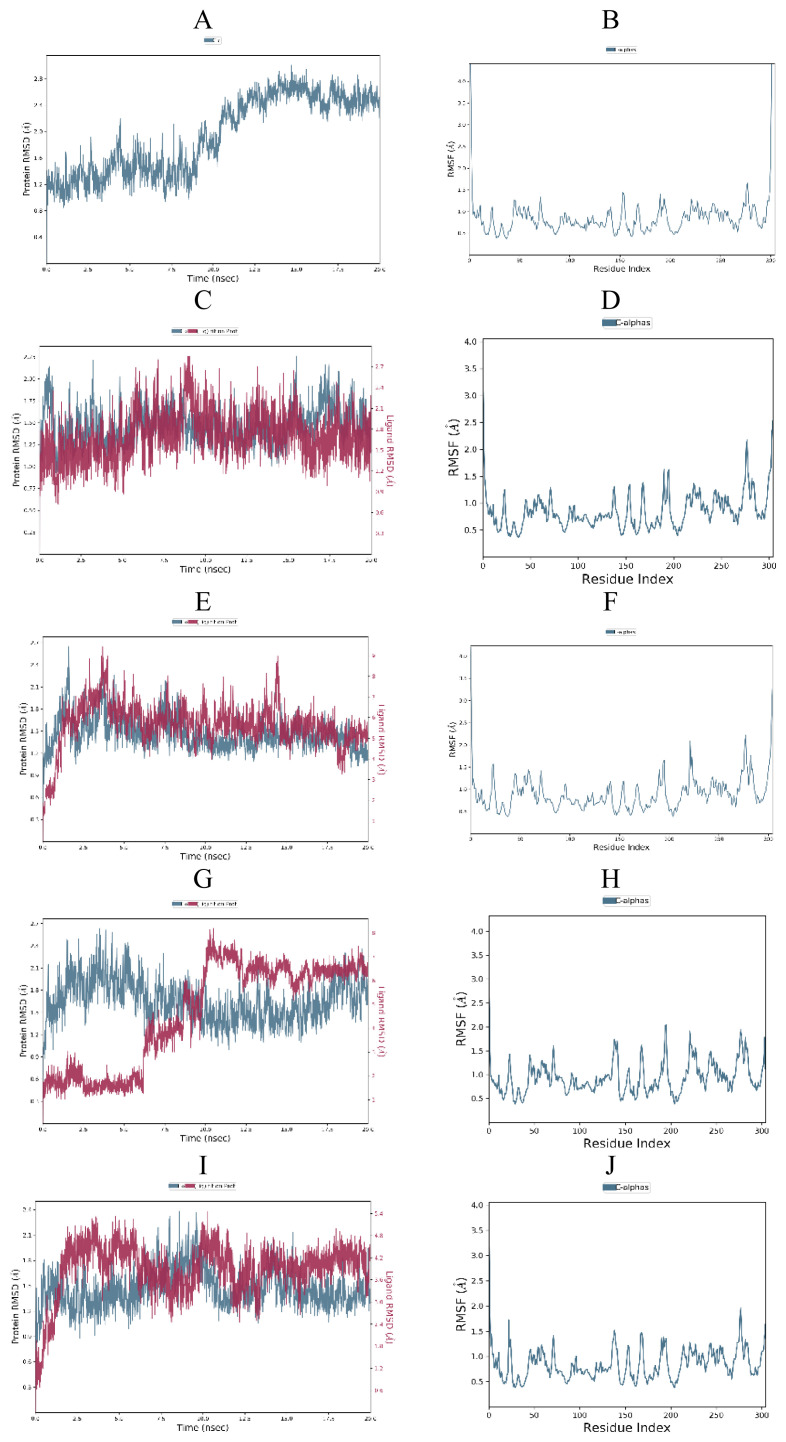
RMSD and RMSF analysis of MD simulation trajectory obtained for: (A,B) free protein (6W63), (C,D) protein-cocrystallized ligand complex, (E,F) protein-compound **6** complex, (G,H) protein-compound **8** complex, (I,J) protein-compound **17** complex.

To investigate the stability of the free SARS-CoV-2 Mpro (PDB ID: 6W63, 6XHM and 7D1M), protein-cocrystallized ligand complexes and protein-top three docked ligand complexes, MD simulations were carried out for 20 ns. Obtained RMSD and RMSF plots for free proteins, protein-cocrystallized ligand complexes, and protein-docked compound complexes are shown in Figures 13–15.

The RMSD plot of free SARS-CoV-2 Mpro (6W63) indicated that protein stabilized shortly after beginning the simulation, then raised and reached maximum of 2.98 Å at 14.70 ns (Figure 15A). On the other hand, the RMDS plots of the SARS-CoV-2 Mpro (6XHM and 7D1M) revealed that proteins remained consistent for almost the entire simulation with peaks reached 2.5 Å at 17.5 ns and 2.49 Å at 5.05 ns, respectively (Figures 13A and 14A). Moreover, no significant deviation in the RMSF plot was observed during the simulation (Figures 13B and 15B), except in the case of SARS-CoV-2 Mpro (7D1M), where the highest fluctuation was observed in the Glu47 and Leu50 with RMSF values of 2.46 and 2.50 Å, respectively (Figure 14B).

In the SARS-CoV-2 Mpro-cocrystallized ligand complex (6XHM), protein was stable till the very end of the simulation, while ligand showed a little fluctuation at the beginning of the simulation with value of the RMSD slightly above the 2 Å, and became stabilized after 10.58 ns (Figure 13C). The RMSF of this complex was stable with fewer fluctuations observed in the Tyr154, Arg222, and Asn277 (Figure 13D).

The RMSD plot of the SARS-CoV-2 Mpro-cocrystallized ligand complex (7D1M) indicated that protein was very stable with RMSD of 2.22 Å at 15.82 ns, while K36 demonstrated a little fluctuation at 6.05 ns with RMSD value of 3.1 Å and further got stabilized after 12.5 ns (Figure 14C). The RMSF of the complex remained less than 2 Å throughout the 20 ns simulation (Figure 14D).

For the SARS-CoV-2 Mpro-cocrystallized ligand complex (6W63), the protein attained a maximum RMSD value of 2.20 Å at 15.47 ns and remained relatively constant until the end of the simulation. In case of ligand RMSD, a slight divergence can be seen with X77 reaching maximum of 2.85 Å at 9.03 ns (Figure 15C). The RMSF of this complex reached 2.3 Å throughout the simulation with the highest fluctuation observed in the Gly278 with RMSF of 2.18 Å (Figure 15D).

The protein (6XHM) RMSD in the SARS-CoV-2 Mpro-compound **6** complex was stable throughout the simulation with insignificant fluctuations implying that the protease has not undergone large conformational changes. On the other hand, RMSD plot revealed that ligand demonstrated very similar trend as protein with maximum observed slightly above 2 Å (Figure 13E). Very similar to the reference protein-cocrystallized ligand complex, the RMSF remained less than 2 Å throughout the entire simulation indicating a stable protein-ligand complex (Figure 13F). As we can see in Figure 13G, protein (6XHM) in SARS-CoV-2 Mpro-compound **8** complex showed very little fluctuation and reached peak after 12.5 ns (2.52 Å), while ligand was relatively stable till 8.02 ns, then got a sharp jump with RMSD values above 8 Å, and finally became stable. The highest fluctuations in RMSF plot were observed in the Arg222 and Gly278 with RMSF values of 2.0 and 1.82 Å, respectively (Figure 13H). For the SARS-CoV-2 Mpro-compound **17** complex, the protein (6XHM) RMSD value grew steadily, reaching a peak of nearly 4 Å. A similar trend was observed in RMSD plot of ligand, where after initial fluctuation, the maximum deviation of 3.82 Å was observed at 9.98 ns (Figure 13I). The RMSF of the complex was higher than 2 Å with maximum fluctuation observed in the residue Gly278 (Figure 13J).

In the RMSD protein (7D1M) plot of the SARS-CoV-2 Mpro-compound **6** complex a slight divergence can be seen towards the end of the simulation with maximum reached at 2.35 Å. On the other hand, ligand **6** demonstrated a very sharp jump in RMSD value from 0.94 Å to 8.07 Å, and later was stabilized after 6 ns (Figure 14E). The RMSF of the complex was lower than 2 Å during simulation with maximum fluctuation observed in the residue Gly278 (Figure 14F). In the SARS-CoV-2 Mpro-compound **8** complex, the protein (7D1M) attained a maximum RMSD value of 2.18 Å at 13.97 ns and remained relatively consistent till the end of the simulation. At the beginning of the simulation, the ligand showed a slight increase in RMSD values, then slightly fluctuated and became stable with 4.88 Å maximum at 16.72 ns (Figure 14G). The RMSF of the complex was lower than 2 Å during simulation with maximum fluctuation observed in the residues Asn274 and Gly278 (Figure 14H). There were very little observed fluctuations of the protein (7D1M) and ligand in the SARS-CoV-2 Mpro-compound **17** complex during MD simulation. Protein was very stable with RMSD values lower than 2 Å during the entire simulation. In addition, the ligand demonstrated minimal fluctuations with RMSD value of 2.82 Å at 15.42 ns (Figure 14I). The RMSF of the complex was lower than 2 Å during whole simulation with maximum deviation observed in the residue Gly278 (Figure 14J).

There were no much-observed fluctuations in the SARS-CoV-2 Mpro-compound **6** complex during MD simulation. Protein (6W63) in this complex underwent fluctuations initially and reached 2.58 Å at 1.61 ns, further decreased, and became stable. In the RMSD plot of the ligand, the fluctuation was observed during the first 3.69 ns of trajectory with RMSD maximum of 8.67 Å. Furthermore, the ligand RMSD stabilized until 14.44 ns, then reached a new maximum of 8.34 Å, and then stabilized again (Figure 15E). The RMSF plot revealed almost identical trend as in the case of reference protein-cocrystallized ligand complex (Figure 15F). The RMSD plot of SARS-CoV-2 Mpro-compound **8** complex showed that protein (6W63) was stable until 6 ns, then decreased slightly, and became stabilized after 10 ns. The ligand was also stable until 6 ns of simulation within RMSD value of 1.8 Å and then reached RMSD maximum of 8.18 Å at 10.48 ns (Figure 15G). The highest RMSF value of 2.04 Å was observed towards Thr196 residue (Figure 15H). Protein (6W63) in the SARS-CoV-2 Mpro-compound **17** complex showed stability till 7.5 ns with RMSD value of 2.24 Å at 8.58 ns, then slightly fluctuated, and became stable after 15 ns with RMSD value near 2.0 Å. RMSD plot of the ligand revealed little fluctuations with initial maximum deviation observed at 3.60 ns (4.53 Å) that later got stabilized after 13.47 ns (Figure 15I). The maximum fluctuation in RMSF (1.97 Å) was observed in the residue Gly278 (Figure 15J).

**Figure 16:**
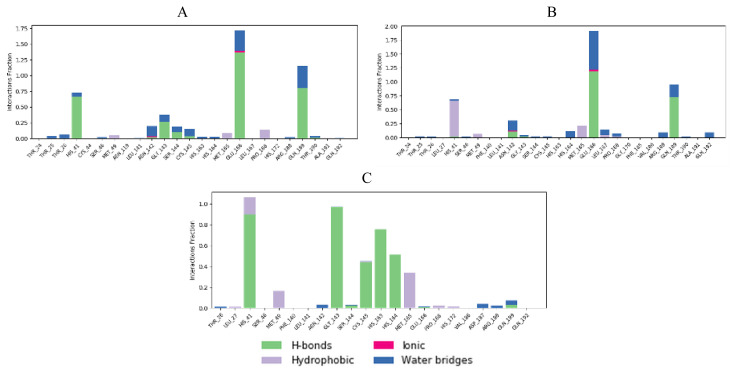
Protein (6XHM)-ligand contacts histogram during MD simulation for: (A) protein-compound **6** complex, (B) protein-compound **8** complex, (C) protein-compound **17** complex.

**Figure 17:**
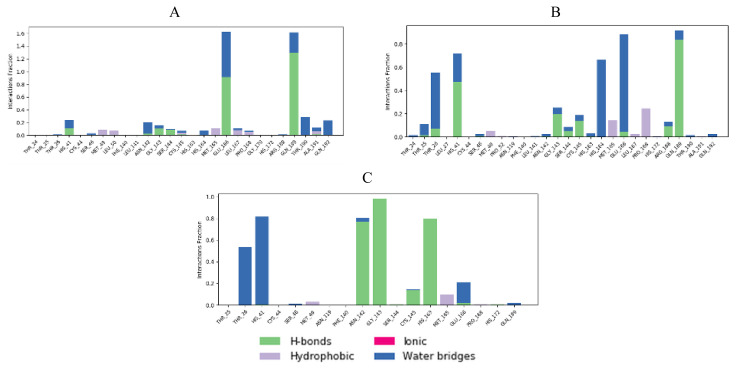
Protein (7D1M)-ligand contacts histogram during MD simulation for: (A) protein-compound **6** complex, (B) protein-compound **8** complex, (C) protein-compound **17** complex.

**Figure 18:**
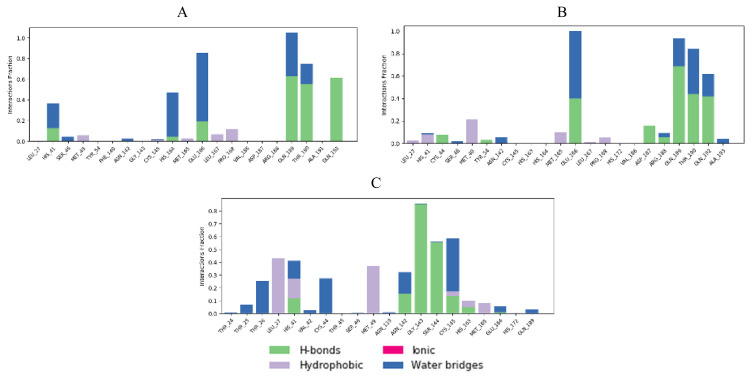
Protein (6W63)-ligand contacts histogram during MD simulation for: (A) protein-compound **6** complex, (B) protein-compound **8** complex, (C) protein-compound **17** complex.

Interaction fractions of residues involved in protein-ligand contacts during MD simulations provide a deeper insight in terms of studying conformational stability (Figures 16–18).

### 3.6. MM/GBSA calculations

MMGBSA approach was used to calculate the average free binding energy. The ΔG bind parameters of cocrystallized ligands and top three docked compounds are given in Table 7.

Scheme illustrates the methodological principle for selecting compounds with the highest binding affinity.

## 4. Discussion

The aim of the present study was to provide a basis for experimental research from the perspective of protein-ligand interactions. In this study, various ligands were identified from PubChem database based on the structure of SARS-CoV-2 main protease inhibitors. Three-dimensional similarity search of three initial compounds led to the set of 18 molecules. Ten of these compounds showed nontumorigenic and nonmutagenic properties, as well as good intestinal absorption empirically estimated using Lipinski’s and Egan’s rules.

The crystal structures of the SARS-CoV-2 main protease with PDB codes 6W63, 7D1M and 6XHM were selected for docking analysis. Cocrystal molecules X77, K36, and V2M are ligand molecules used as the binding site control. Since the cocrystal ligands bind to the target proteins with high affinity, their interactions with amino acid residues are expected to be essential for effective ligand binding into the active site of protein. Therefore, if tested molecules establish a numerous interactions with these key binding residues, they could effectively inhibit the function of target enzyme. Type, number of key binding interactions, and corresponding docking scores presented the three main criteria for assessing the binding affinity of the tested compounds towards SARS-CoV-2 main protease. Based on the binding affinity analysis, among tested molecules, we selected **6**, **8**, and **17** as compounds with the highest potential for SARS-CoV-2 main protease. Their binding modes will be further discussed in detail. MD simulations were then carried out on these three top docked compounds to get a deeper understanding of the compounds affinity for SARS-CoV-2 Mpro active site. Protein-ligand complexes were then subjected to MM/GBSA calculations to estimate average binding free energies.

On the other hand, compounds **3**, **4**, **9**, **10**, **11**, **12**, and **13** bound to the target proteins with lower affinity. We made this conclusion based on the type and number of significant binding interactions with special reference to the number of key hydrogen bond interactions, as well as the docking scores of the tested compounds for all of the three target proteins. If we consider only the docking scores for all of three targets, compound **9** showed the highest binding affinity towards 6W63, 6XHM, and 7D1M. However, during molecular fitting of compound **9** to all three target proteins, it established only a few hydrogen bond interactions. Similarly, compounds **4**, **11**, **12**, and **13** formed only one significant hydrogen bond interaction. Although compound **6** bound to the 6W63 achieving one significant hydrogen bond, this compound bound to the other two targets forming with each of them five key hydrogen bond interactions. For these reasons, this compound was classified as a molecule with higher affinity. Futhermore, **3** and **10** had a relatively few significant hydrogen bond interactions compared to the compounds with a higher binding affinity. In order to assess binding affinities of tested compounds, we also determined the binding energies of the cocrystal ligands.

When cocrystal V2M ligand bound to the target protein, the molecule achieved free binding energy value of −7.2 kcal/mol. On the other hand, compounds **6** and **8** bound to this target with a higher binding energies of −6.9 and −6.6 kcal/mol, while molecule **17** achieved lower docking score of −7.4 kcal/mol. MM/GBSA calculation confirmed these findings, since compound **17** also demonstrated the lowest average ΔG binding energy in comparison to compounds **6** and **8**. In contrast to docking results, compound **17** showed higher ΔG binding energy than cocrystallized ligand V2M (Table 7). In the crystal structure of the 6XHM, cocrystal V2M formed a covalent bond with Cys145, hydroxyl group of carbinol created hydrogen bond with Cys145, while primary hydroxyl group of carbinol interacted with His41. In the S1 subsite, lactam carbonyl formed hydrogen bond with His163, while the NH group of lactam interacted with Glu166. The NH group of isobutyl residue and Gln189 formed a hydrogen bond, while P1 NH accomplished hydrogen interaction with His164. Finally, at S2 subsite, the V2M interacted with residues Asp187, Arg188, Glu189, Met49, and His41 [48]. In the active site of 6XHM, compound **6** formed identical multiple hydrogen bond interaction between residue Glu166 and donor nitrogen atom of indole ring and adjacent oxygen atom of the carboxamide group near the S1 subsite. Similar multiple hydrogen bond interactions with residue Glu166 were observed during the binding of the rosuvastatin to the SARS-CoV-2 main protease [55]. Compound **6** also created two additional hydrogen bond interactions with Gln189 at S2 subsite through the donor nitrogen atom of carboxamide group and oxygen atom of the dimethylcarbonyl group. This molecule formed one additional hydrogen bond with His163 at S1 subsite. At S2 subpocket the compound **6** formed hydrophobic interaction with His41 and one more with Met165 at S4 subsite (Figure 5). Similar to compound **6**, lopinavir formed identical hydrogen bond interactions with residues Glu166 and Gln189 and hydrophobic interactions with residues Met164 and His41 [56]. Interaction fractions chart (Figure 16A) shows that the important hydrogen bonds observed in the docked pose of compound **6** (Glu166 and Gln189) did not change during the MD simulation and retained for more than 80% of the simulation time. The overall RMSD plot of SARS-CoV-2 Mpro-compound **6** complex (Figure 13E) showed that compound **6** and protein (6XHM) backbone were lying over each other, indicating the formation of a stable protein-ligand complex. Carbamate oxygen atom of the compound **8** created the key hydrogen bond with residue His41 near S2 subsite, while carbonyl adjacent to isobutyl group of **8** acted as an acceptor of the hydrogen bond in the interaction with His163 near the S1 subsite. At the S2 subsite, the compound **8** formed only one hydrophobic π-alkyl interaction with residue Met49 (Figure 8). In the docking analysis of the Samant et al. [57], quinine also achieved the key bond interactions with His41 and Van der Waals contact with His163. The overall RMSD plot of SARS-CoV-2-Mpro compound **8** complex (Figure 13G) shows minimal mutual fluctuations between ligand and protein (6XHM), so the formation of a complex cannot be ruled out. Compound **17** formed three significant hydrogen bonds between imidazole ring and His164 near the S2 subsite, adjacent acceptor oxygen atom of carboxamide and Cys145, while pyridine ring of **17** near the S1 subsite acted as hydrogen bond acceptor in the interaction with His163. Binding mode of the lovastatin illustrates the similar binding pattern which is reflected through the same hydrogen bond interactions with residues His164 and His163 [55]. At S2 subsite compound **17** formed two key hydrophobic π-alkyl contacts between imidazole ring and resides His41 and Met49 (Figure 11). Very similarly, mefloquine achieved four of five key interactions accomplished by compound **17** (His41, Met49, His164, and Cys145) [57]. In addition, from the interaction fractions bar (Figure 16C), we clearly see that four significant hydrogen bond observed in the best docked pose of compound **17** (His 163, Cys 145, His 164, and Gly 143) remained stable during simulation. Gly143 was dominant interaction which lasted approximately 97% of simulation time. Hydrophobic interaction was also observed with Met49 which lasted approximately 17% of the simulation time. Since the RMSD plots of compound **17** and protein backbone were lying over each other, we can conclude that the ligand is stably bound to viral protease forming a stable complex (Figure 13I).

Cocrystal K36 redocked into the active site of 7D1M with docking score value of −7.3 kcal/mol, achieving hydrogen bond interactions with residues Gln189, Glu166, His163, Phe140, His41, Ser144, Met165, and Gly143 [4]. In comparison, tested compounds **6** and **17** demonstrated equal binding energy value, while compound **8** accomplished the same docking score as cocrystal. However, if we consider the MM/GBSA calculations, we can conclude that compound **17** showed the highest binding affinity towards viral protease in comparison to compounds **6** and **8** and cocrystallized ligand too (Table 7). In addition, compound **17** accomplished a lower MM/GBSA binding energy than cocrystallized ligand K36, indicating a higher binding affinity of this compound in comparison to K36. In the 7D1M complex, the bisulfite group of K36 was removed and molecule created a covalent bond with Cys145. The pyrrolidone ring fit into the S1 pocket and formed hydrogen bond interactions with carboxyl group of Glu166. The isobutyl residue fit into the S2 hydrophobic subpocket which consisted of Arg40, His41, Met49, Tyr54, and Asp187 [4]. During molecular fitting of compound **6** key binding interaction residue Glu166 formed only weak carbon hydrogen bond with methoxyindole and dimethylcarbonyl residue instead of conventional hydrogen bond which were formed by cocrystal ligand. Nitrogen donor atom of the carboxamide adjacent to indole ring formed hydrogen bond with Gln189 residue near S2 subsite, while aldehyde residue of the **6** acted as hydrogen bond acceptor in the interaction with His41 at S1′ subsite. Moreover, residue Ser144 interacted with carbonyldimethylamino group of the inhibitor, while Cys145 residue achieved multiple hydrogen interactions with donor nitrogen atom from isobutylamide group and oxygen atom from carbonyldimethylamino residue. At the same time, indole ring of the inhibitor occupied a S4 pocket site by forming a π-alkyl interaction with Met165 and isobutyl residue formed π-σ interaction with His41 (Figure 6). Ciprofloxacin formed molecular interactions with four of the six key binding residues (Ser144, Cys145, Glu166, and His41) accomplished by compound **6** [57]. Figure 17A indicates that three significant hydrogen bonds (Gln189, His 41, and Ser 144) of the docked pose were retained from the best docked pose of compound **6** during the simulation time of 20 ns. Interaction with Gln189 remained stable for near 130% of the simulation time due to the formation of multiple subtypes interactions. Hydrophobic contact was also observed with Met165 residue that lasted approximately 11% of simulation time. However, new interactions of water bridges type were also established with residues Ala191 and Thr190 that last approximately 10% of the simulation. The RMSD plot (Figure 14E) indicated a stable protein-ligand complex in the last 15 ns of simulation period. Molecular docking analysis revealed that compound **8** formed seven key hydrogen bond interactions (Figure 9). Benzylcarbamate residue of **8** created a double hydrogen bonding interaction with Gln189 residue near S4 pocket site, while Glu166 residue acted as hydrogen bond donor in the interaction with carbamate carbonyl of the **8** at S1 subsite. Atorvastatin formed identical multiple hydrogen interaction with residue Gln189 [55]. Leucine P2 carbonyl of the **8** interacted with His41 by forming conventional hydrogen bond. Oxoethanol carbonyl of **8** also created three key hydrogen bond interactions with residues Cys145, Ser144, and Gly143 near the S1 subsite. Leucine residue occupied S2 subsite in which side chain of the amino acid formed hydrophobic π-alkyl interaction with Met49. Except for residue Met49, nelfinavir [56] also formed bond interactions with the same key residues Gln189, Glu166 (hydrogen bonds), Met165 (π-S), His41 (π-π), Cys145 (alkyl), and Ser144 (Van der Waals contact). As we can see from Figure 17B, even six significant hydrogen bond interactions with residue His41, Cys145, Ser144, Gln189, Gln189, and Glu166 were retained from the best docked pose of the compound **8** during MD simulation. However, Gln189 showed interaction approximately 84% of the simulation time. One significant hydrophobic interaction with Met49 residue was also observed which lasted 5% of simulation time. Figure 14G shows that RMSD plots of compound **8** and SARS-CoV-2 Mpro (7D1M) overlap each other, so we can conclude that the ligand is stably bound to protease binding site and has not diffused away from the bound position. Compound **17** showed high affinity towards target enzyme due to six key hydrogen bonds achieved (Figure 12). Carbonyl adjacent to isobutyl residue of the **17** fit S2 subsite to form hydrogen interaction with His41. P1 imidazole ring of the **17** created hydrogen bonds with Asn142 thereby acting as donor and with His163 acting as acceptor. Residue Cys145 formed two hydrogen bonds with isobutylamide group and carbonyl of imidazolecarboxamide, while Gln189 residue created one hydrogen bond interaction with nitrogen donor atom of isobutylamide group. Near the S1′ subsite compound **17** formed π-alkyl hydrophobic interaction with Met49. These residues, Gln189 and Asn142, were also identified as important binding residues in recent docking study by Yu et al. [58]. The results showed that luteolin formed five hydrogen bonds with above-mentioned residues, Leu4 and Thr26 [58]. Three key hydrogen bond interactions (His163, Cys145, and Asn142) and one hydrophobic contact (Met49) were retained from the best docked pose of the compound **17** during MD simulation. His163 was dominant interaction which lasted more approximately 80% of simulation time. Residue His41 and Gln189 were engaged in the water bridges formation. The hydrophobic π-alkyl contact between pyridine ring of the compound **17** and Met49 was also observed. The RMSD plot (Figure 14I) indicated a stable ligand-protein complex throughout the entire simulation period with RMSD fluctuation values of 0.8 Å for both the protein and ligand.

Docking analysis revealed that cocrystal ligand X77 bound to the 6W63 with docking score of −8.3 kcal/mol. In comparison, top compounds demonstrated higher docking score values of −7.1, −6.9, and −8.1 kcal/mol, while compound **17** established the highest number of key hydrogen interactions. MM/GBSA calculations (Table 7) confirmed our previous conclusion that all three top docked compounds demonstrated the lower binding affinity in comparison to cocrystallized ligand X77. Among the best docked compounds, in terms of average binding energy, compound **17** stands out as molecule with the highest potential towards target protein. The binding site of cocrystal X77 ligand in the target protein structure included four amino acid residues involved in the hydrogen bonding (Glu166, Gly143, Asn142, and His163). The strongest binding occurred at Gly143 in which two hydrogen bonds were formed between oxygen atom of 1*H*-imidazole-4-carboxamide and nitrogen atom of the imidazole residue. Besides, pyridine donor nitrogen atom of the X77 formed hydrogen bond with His163 residue, oxygen atom of 1*H*-imidazole-4 carboxamide with Asn142, while the residue Glu166 interacted with oxygen atom of the cyclohexylamino inhibitor residue. Compound **6** achieved only one key hydrogen bond interaction between carbonyldimetylamino residue and Gln189 at S4 subsite. Weak carbon hydrogen interaction was observed between dimethylamino residue and Glu166. Isobutyl residue of leucine formed hydrophobic π-alkyl interaction with residue His41 near the S1′ subsite. The remaining contacts were hydrophobic amide-π stacked interaction with Leu141 at S1 subsite and Van der Waals force with Asn142 (Figure 4). Interaction fraction diagram for the SARS-CoV-2 Mpro-compound **6** complex contacts shows that one hydrogen bond interaction (Gln189) retained from the docked pose of the selected compound during MD simulation. On the other hand, residues Asn142, Glu166, and His41 of 6W63 that formed hydrophobic contacts in the docked pose of ligand, now form the water bridges during MD simulation. Overall RMSD plot of the SARS-CoV-2 Mpro-compound **6** complex (Figure 15E) shows very good mutual overlapping of the ligand and protein, implying the formation of stable protein-ligand complex. During the binding process of compound **8**, three hydrogen bond interactions were observed (Figure 7). Near the S2 subsite amino acid residue Gln189 interacted with leucine residue, while residue Glu166 formed conventional hydrogen bond with benzylcarbamate nitrogen donor atom of the **8** near the S4 subsite. Moreover, hydroxyl group of the oxoethanole residue formed hydrogen bond interaction with residue Cys44. At the S1′ subsite, hydrophobic interactions were formed between isobutyl side chain of the leucine and His41 and Cys44 residues. Weak carbon hydrogen interaction was formed between carbonyl of the same isobutyl group and Met49. Additionally, hydrophobic interaction was created between Met165 and side chain of the leucine near the S4 subsite. During MD simulation, seven significant bond interactions retained from the docked pose of compound **8** in the active site of SARS-CoV-2 Mpro (6W63). There were three hydrogen bonds (Glu 166, Gln189 and Cys44) and four hydrophobic interactions (Met165, Met49, His41, and Pro168). Gln189 was dominant hydrogen bond interaction that lasted approximately 69% of simulation time. Since the RMSD plots of compound **8** and protein backbone were not lying over each other, we can conclude that no stable protein-ligand complex was formed (Figure15G). Docked pose of the compound **17** showed that four binding subpockets of the SARS-CoV-2 main protease were well occupied. High affinity of the **17** is a consequence of four hydrogen bonds formed between nitrogen atom acceptor of pyridine ring and His163 near the S1 subsite, keto group of 1*H*-imidazole-5-carboxamide and Gly143 at S1′ subsite, donor nitrogen atom from imidazole and Cys145 also near the S1′ subsite, while Glu166 residue interacted with leucine residue near the S4 subsite. Near the subsites S1 and S1′, compound **17** formed three additional carbon hydrogen interactions with Phe140, Asn142, and Leu141 residues. Compound **17** formed additional two key hydrophobic interactions with Met49 (π-sulphur) and His41 (π-π) at S2 subsite (Figure 10). On the other hand, remdesivir formed hydrogen bonds (Glu240, His246, and Pro48) and hydrophobic interactions (Pro132, Ile200, and Gln107) with different amino acid residues into the SARS-CoV-2 main protease active site [58] suggesting the different binding mode of this antiviral drug compared to our tested compounds. On the contrary, results of the recent docking study revealed that remdesivir formed two hydrogen bond interactions with residues Phe294 and Gln110, which also were not observed during molecular fitting of our compounds [3]. Interaction fraction chart (Figure 18C) shows that all four significant hydrogen bond interactions (Glu166, Gly143, His163, and Cys49) observed in the top docked pose of the compound **17** prevailed during MD simulation. Residue Cys145 and His41 were also involved in forming water bridged and hydrophobic interactions during MD simulation, while residue Met49 engaged in π-sulfur contact with tert-butylbenzene of the compound **17**. Since the RMSD plots of compound **17** and protein were lying over each other throughout simulation, we can conclude that a highly stable complex was formed that did not undergo large conformational changes (Figure 15I).

If we compare the binding affinity of our compounds with the binding affinity of in silico tested antiviral drugs, contradictory conclusions can be made. Narkhede et al. examined the binding interactions of the different antiviral drugs with SARS-CoV-2 main protease, whereby binding energies were in the range of −4.7 to −7.3 kcal/mol, which were significantly higher than the values obtained in this study. Oseltamivir, ritonavir, and ribavirin bound to the target protein with binding energy values of −7.3, −6.5, and −5.4 kcal/mol, respectively [3], indicating a lower affinity of these compounds compared to those tested in this study. In two different interaction studies [3,59] conducted in AutoDock Vina and AutoDock, remdesivir demonstrated higher docking scores of −6.5 and −5.51 kcal/mol in comparison to tested compounds. MM/GBSA binding energies of compound **17** obtained in this study (−76.44, −67.88, and −62.54 kcal/mol) are far lower in comparison to indinavir and remdesivir (−58.16 and −36.15 kcal/mol), indicating a lower affinity of these compounds compared to tested compound **17** [60]. Different classes of antibiotics were also tested against main protease of SARS-CoV-2, such as azithromycine [61], clindamycine, and ciprofloxacin [57]. These compounds showed free energy values of −6.3, −7.4, and −7.5 kcal/mol, respectively, which was comparable to the values obtained for our compounds. Pham et al. [62] examined the binding mode of possible inhibitors from ZINC15 subdatabase against SARS-CoV-2 main protease. Among them, Sennidin A, Guamecycline, and Uralsaponin A had significantly higher binding affinities in comparison to our compounds. Keretsu et al. [63] performed molecular docking analysis in different program packages in order to identify potential SARS-CoV-2 main protease inhibitors from protease inhibitors database MEROPS. In this in silico study, the best docking score achieved saquinavir with binding energy of −9.1 kcal/mol, which was also a lower value than binding score of compound **17**. Artemisinin and its derivatives artesunate and artemether bound to the SARS-CoV-2 main protease with binding energy values of −7.5, −7.9, and −7.7 kcal/mol [63], respectively, which was also comparable to our findings. The use of hydroxychloroquine in the COVID-19 infection treatment was questionable due to controversial study results. However, on 17 June 2020, WHO announced that the hydroxychloroquine trial was being stopped [64]. Based on the evidence from the Cochrane review [65], hydroxychloroquine does not cause the mortality reduction of hospitalized COVID-19 patients in comparison to standard care. Nevertheless, results of two different docking studies [3,61] showed that the binding energies of hydroxychloroquine were −5.3 and −5.0 kcal/mol, which were significantly higher than free binding energies of our top compounds. Simvastatin and pitavastatin bound to the viral protease with free energies −7.9 and −8.2 kcal/mol [55], which were almost identical to binding affinities obtained for our best docked compounds **6**, **8**, and **17**. In a recent study, Alnajjar et al. obtained MM/GBSA average binding energies for the angiotensin II receptor blockers. Fimasartan and candesartan showed ΔG bind values of −50.33 and −53.05 kcal/mol, which are comparable to calculated ΔG bind values for tested compounds **6** and **8**. However, compound **17** demonstrated significantly higher binding affinity in comparison to mentioned sartans with calculated ΔG bind value that was in range of −62 to −76 kcal/mol [66].

In a recent docking study, binding analysis revealed that interactions of the compounds with S1 subsite residues were crucial for stable interactions with target enzyme [63]. Thus, all three potential inhibitors **6**, **8**, and **17** formed hydrogen bond interaction with His163 during molecular fitting into the 6XHM active site, while compound **6** created additional multiple hydrogen bond interaction with Glu166 near the S1 subsite. On the other hand, compound **8** formed even four hydrogen bond interactions during molecular fitting into the 7D1M active site with residues Glu166, Gly143, Ser44, and Cys145 at S1 subsite. Results of the above-mentioned docking study [63] demonstrated that compounds which formed hydrogen bonds and hydrophobic interactions at the S4 subsite showed also relatively high binding energy values, suggesting the importance of these interactions in enzyme inhibition. Compound **8** showed such binding pattern during molecular fitting into the 6W63. Namely, compound **8** formed hydrogen bond interaction with Glu166 and hydrophobic interaction with Met165. In line with our results, Keretsu et al. [63] also observed that substituents which formed hydrophobic interactions at the S1′ subsite were also important for binding to SARS-CoV-2 main protease. These hydrophobic contacts at S1′ have been also observed in the interactions of compound **6** and 6W63. Similarly, compound **8** bound to the 6W63 and formed three hydrophobic interactions with residues Cys44, His41, and Met49. From these observations, we can assume that substituents capable of forming hydrophobic and hydrogen bond interactions with S1′ residues may increase the binding affinity. Consequently, having both of these types of interactions with target enzyme can closely mimic the substrate binding pattern. Compound **17** showed such binding pattern during the molecular fitting into the 6W63 structure achieving two hydrogen interactions with residues Gly143 and Cys145 and one hydrophobic interaction with Asn142 near the S1′ subsite.

## 5. Conclusion

Similarity search of three initial SARS-CoV-2 main protease inhibitors led to the set of 18 molecules obtained from PubChem database. Ten of these compounds showed nontumorigenic and nonmutagenic properties, as well as good intestinal absorption empirically estimated using Lipinski’s and Egan’s rules. The present study confirmed the affinities of the tested compounds against SARS-CoV-2 main protease. Based on type, number of key binding interactions and corresponding docking scores, we concluded that compounds **6**, **8**, and **17** bound to the target proteins with the highest affinity. Additionally, top three docked compounds were subjected to MD simulation studies to examine the stability and flexibility of their complex with viral protease. The MD simulation results indicated that protein-top docked ligand complexes have shown good conformational stability throughout the 20 ns of simulation. Among the top ranked molecules, compound **17** emerged as the best in silico hit based on the docking score, MM/GBSA binding energy and MD results. Although the biological activity of these in silico hits has yet to be determined, it is expected that they could serve as suitable lead molecules for the development of new antiviral drugs against COVID-19 pandemic.

## Figures and Tables

**Scheme:**
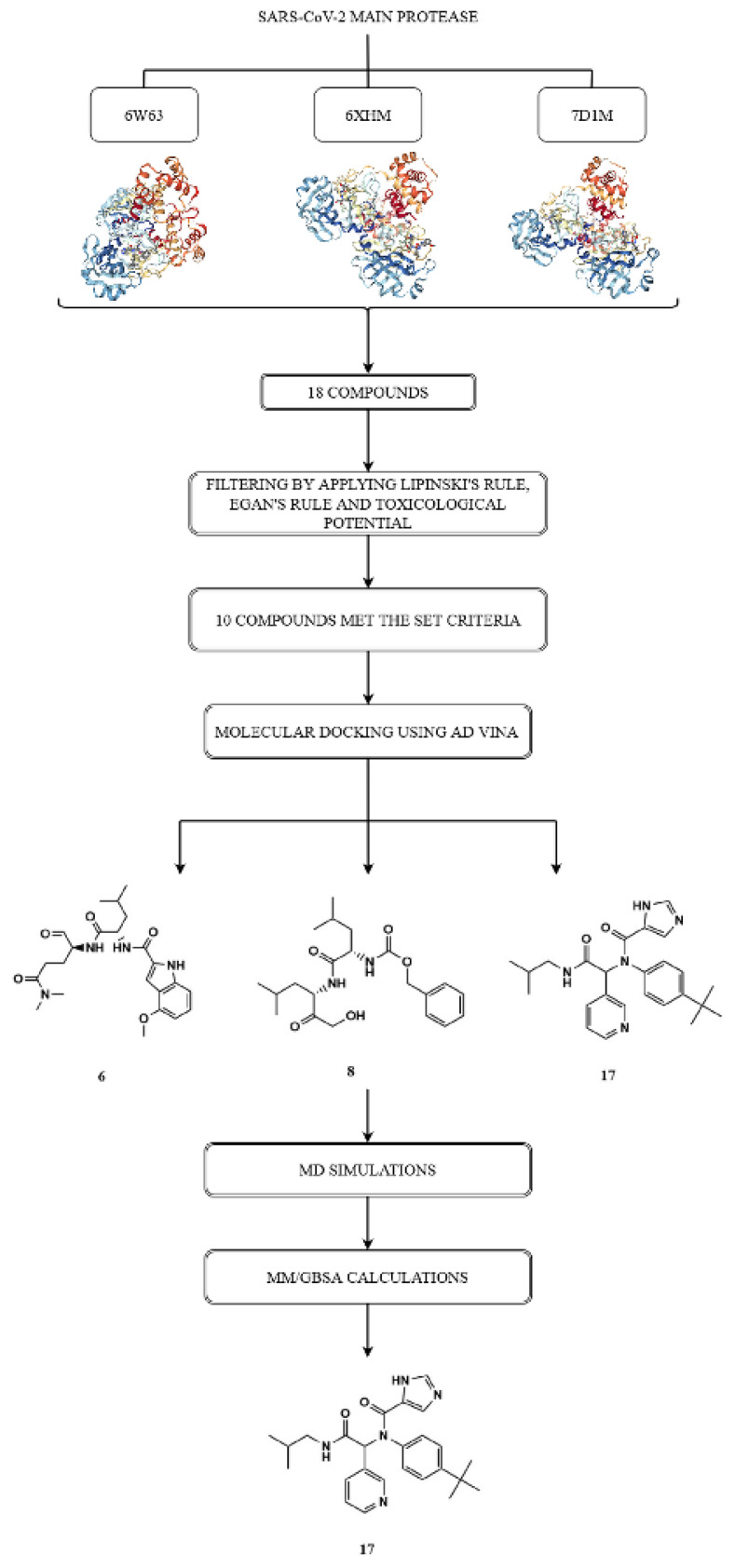
A stepwise approach in selection compounds with the highest binding affinity.

**Table 1:** Protein targets selected for the study.

Target	Selected PDB (resolution)	Cocrystallized ligand	Chains	Selected chain
SARS-CoV-2 main protease	6W63 (2.10 Å)	*N*-(4-tert-butylphenyl)-*N*-[(1*R*)-2-(cyclohexylamino)-2-oxo-1-(pyridin-3-yl)ethyl]-1*H*-imidazole-4-carboxamide (X77)	A	A
SARS-CoV-2 main protease	6XHM (1.41 Å)	*N*-[(2*S*)-1-({(2*S*)-4-hydroxy-3-oxo-1-[(3*S*)-2-oxopyrrolidin-3-yl]butan-2-yl}amino)-4-methyl-1-oxopentan-2-yl]-4-methoxy-1*H*-indole-2-carboxamide (V2M)	A, B	A
SARS-CoV-2 main protease	7D1M (1.35 Å)	(1*S*,2*S*)-2-({*N*-[(benzyloxy)carbonyl]-*L*-leucyl}amino)-1-hydroxy-3-[(3*S*)-2-oxopyrrolidin-3-yl]propane-1-sulfonic acid (K36)	A, B	A

**Table 2:** Lipophilicity, water solubility, and physicochemical properties of initial compounds.

Ligand number	Molecular formula	Number of heavy atoms	Number of aromatic heavy atoms	Number of rotatable bonds	Molar refractivity	LogP (XLogP3)^a^	LogP (SILICOS-IT)^b^	LogS (SILICOS-IT)	Solubility (mol/L)
1	C_21_H_31_N_3_O_8_S	33	6	14	122.68	0.71	0.44	−3.75	1.76·10^−4^
2	C_24_H_35_N_3_O_8_S	36	6	14	134.99	2.12	0.98	−4.32	4.78·10^−5^
3	C_27_H_40_N_2_O_5_	34	6	15	134.20	5.49	4.67	−5.46	3.47·10^−6^
4	C_23_H_30_N_2_O_4_	29	12	13	112.60	3.84	3.69	−6.54	2.86·10^−7^
5	C_24_H_33_ClN_4_O_5_	34	9	15	131.37	2.90	3.99	−6.41	3.94·10^−7^
6	C_23_H_32_N_4_O_5_	32	9	14	121.77	2.05	3.18	−5.43	3.67·10^−6^
7	C_21_H_29_N_3_O_5_S	30	6	13	119.12	2.41	2.62	−5.04	9.10·10^−6^
8	C_21_H_32_N_2_O_5_	28	6	14	107.55	3.33	3.15	−4.80	1.57·10^−5^
9	C_27_H_33_N_5_O_2_	34	17	9	133.79	4.87	4.28	−7.74	1.81·10^−8^
10	C_26_H_29_N_3_O_3_	32	17	9	124.42	4.92	4.25	−7.73	1.87·10^−8^
11	C_24_H_26_N_2_O_4_S	31	16	9	121.22	4.88	4.58	−6.70	2.00·10^−7^
12	C_23_H_22_FN_3_O_2_S	30	17	8	115.40	4.29	4.75	−7.00	1.00·10^−7^
13	C_24_H_25_N_3_O_3_	30	17	8	114.80	3.94	3.61	−7.07	8.51·10^−8^
14	C_25_H_26_ClFN_4_O_2_S	34	17	8	132.95	6.14	6.12	−8.22	6.07·10^−9^
15	C_24_H_26_N_2_O_3_S	30	16	8	119.85	5.09	5.33	−7.08	8.28·10^−8^
16	C_28_H_33_N_3_O_2_S	34	17	10	139.42	5.50	6.13	−8.18	6.68·10^−9^
17	C_25_H_31_N_5_O_2_	32	17	10	126.29	4.39	4.15	−7.56	2.75·10^−8^
18	C_29_H_34_N_2_O_2_S	34	17	10	141.62	6.53	6.70	−8.55	2.83·10^−9^

a XLOGP program, version 3.3.2. Shanghai Institute of Organic Chemistry.

b FILTER-IT program, version 1.0.2. SILICOS-IT, http://www.silicos-it.com

**Table 3:** Toxicology data and molecular properties of initial compounds shortlisted by implementing Lipinski’s and Egan’s rules.

Ligand number	Molecular weight (g/mol)	LogP	H-bond acceptors	H-bond donors	Lipinski’s rule	Polar surface (Å^2^)	Egan’s rule	Mutagenic	Tumorigenic
1	485.556	−0.9668	11	5	No	179.51	No	No	No
2	525.621	−0.2954	11	5	No	179.51	No	No	No
3	472.624	3.5523	7	3	Yes	104.73	Yes	No	No
4	398.501	3.1268	6	3	Yes	87.66	Yes	No	No
5	493.002	1.9409	9	3	Yes	120.6	Yes	Low	High
6	444.530	1.1712	9	3	Yes	120.6	Yes	No	No
7	435.543	1.0035	8	3	Yes	152.4	No	No	No
8	392.494	2.2134	7	3	Yes	104.73	Yes	No	No
9	459.592	3.7896	7	2	Yes	90.98	Yes	No	No
10	431.534	3.9417	6	1	Yes	75.44	Yes	Low	No
11	438.546	3.9797	6	1	Yes	100.02	Yes	No	No
12	423.511	3.6189	5	1	Yes	90.54	Yes	No	No
13	403.481	3.1841	6	1	Yes	75.44	Yes	No	No
14	501.024	5.4560	6	1	No	103.43	Yes	No	No
15	422.547	4.3955	5	1	Yes	90.79	Yes	No	High
16	475.655	5.0452	5	1	No	90.54	Yes	No	No
17	433.554	3.3129	7	2	Yes	90.98	Yes	No	No
18	474.667	5.9639	4	1	No	77.65	No	No	No

**Table 4:** Molecular docking validation data.

PDB code	Validation of molecular docking	RMSD
6W63	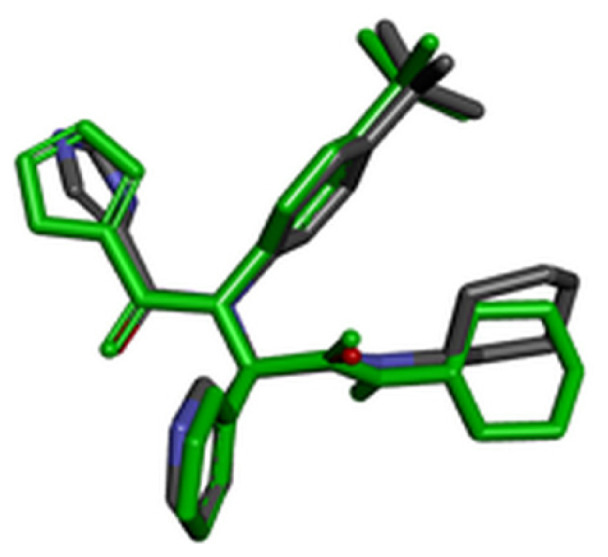	0.9450
6XHM	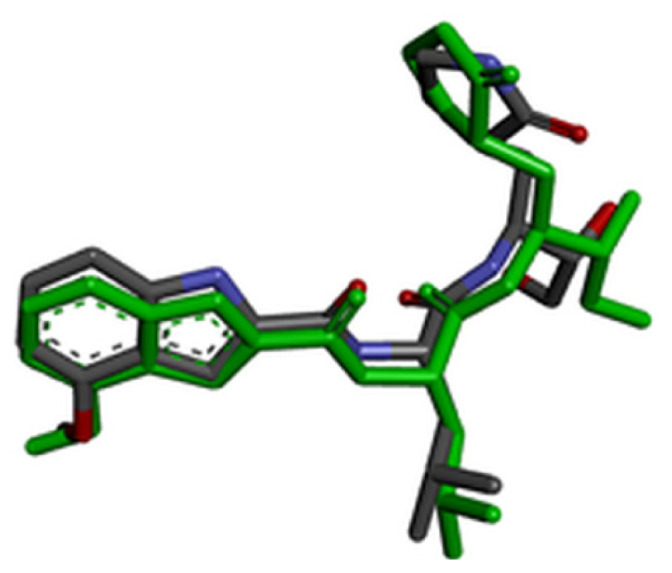	1.2936
7D1M	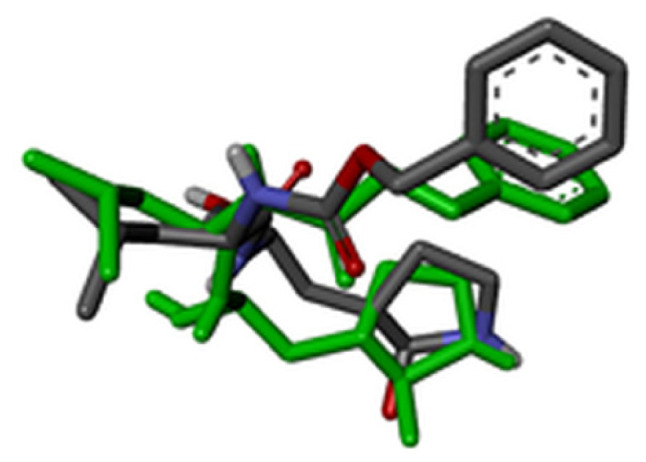	1.7391

**Table 5:** An overview of key binding interactions and docking scores of the compounds with lower binding affinity towards SARS-CoV-2 main protease.

Ligand number	PDB code	Hydrogen bonds		Other interactions	Docking score (kcal/mol)	
	
		Donor/acceptor	Bond length (Å)		Ligand	Cocrystallized ligand

3	6W63	OH/Asn142	2.37	Met165, Asp187, Gln189, Cys44, Met49, His41	−7.0	−8.3
		NH/Glu166	2.94			
		Glu166/O	2.90			
		Glu166/O	1.97			
		His163/O	2.91			

	6XHM	Asn142/O	2.29	Met165, Gly143	−6.8	−7.2
		His163/O	2.23			
		NH/Cys145	2.75			

	7D1M	His41/O	2.84	His163, Leu141, Ser144, Gln189, Glu166	−7.0	−7.1
		Cys145/O	3.38			

4	6W63	OH/Asn142	3.38	Glu166, Met165, Asp187, Gln189, Cys44, Met49, His41	−7.0	−7.4

	6XHM	Glu166/O	1.89	Asn142, Leu141, His41, Met49, Pro168, Ala191, r190	−6.9	−7.2
		NH/Gln189	2.43			
		Gln189/O	2.76			
		NH/Cys145	2.88			

	7D1M	His41/O	2.55	Met165, Leu141, His164, Ser144	−7.2	−7.0
		NH/Gln189	2.05			
		Gln189/O	2.53			
		Glu166/O	2.43			

9	6W63	NH/Glu166	2.55	Asn142, His163, Met49, His41, Pro168	−7.7	−8.3
		Gln189/N	2.65			

	6XHM	His163/N	2.56	Asn142, Leu141, Cys145, Met165, His41, Gln189	−7.7	−7.2
		NH/His164	1.86			

	7D1M	His41/O	2.37	Met165, Leu141, Met49, Asn142, Gln189, Glu166	−8.1	−7.3
		His163/N	2.08			
		NH/His164	1.93			

10	6W63	Glu166/O	2.56	Met165, Cys145, Gln189, Cys44, His41, Pro168	−7.3	−7.9

	6XHM	Gly143/O	2.72	Asn142, Leu141, Cys145, Leu27, Met165, His41, Met49	−7.3	−7.2
		Gln189/O	2.69			

	7D1M	Gln189/O	2.17	His41, Met165, Met49, Cys145, Asn142, Glu166	−7.4	−7.1

11	6W63	Glu166/O	2.50	Met165, Gln189, Met49, His41, Pro168	−7.0	−7.9

	6XHM	NH/His164	2.83	Asn142, Leu141, His41, Met49	−7.2	−7.2
		Cys145/O	3.32			
		Gly143/O	2.75			

	7D1M	NH/His164	2.80	His41, Leu141, Met49, Asn142	−7.3	−7.3
		Cys145/O	3.50			

12	6W63	Gln189/N	2.45	Glu166, Met165, Cys145, Met49, His41	−7.3	−8.3

	6XHM	His163/F	2.38	Phe140, Leu141, Cys145	−6.9	−7.2
		Gln189/O	2.06			

	7D1M	His172/F	3.00	Cys145, Asn142, Gln189, Glu166	−7.7	−7.3
		NH/His164	2.65			

13	6W63	Glu166/O	2.43	Met165, Cys145, Cys44, His41, Pro168	−7.4	−8.3
		Gln189/O	2.34			

	6XHM	Cys145/O	3.79	Asn142, Leu141, Glu166, Met165, His41, Met49, Gln189	−6.6	−7.0

	7D1M	Gln189/O	2.10	His41, Leu141, Met49, Cys145, Asn142	−7.2	−7.0

**Table 6:** An overview of key binding interactions and docking scores of the compounds with higher binding affinity towards SARS-CoV-2 main protease.

Ligand number	PDB code	Hydrogen bonds		Other interactions	Docking score (kcal/mol)	
	
		Donor/acceptor	Bond length (Å)		Ligand	Cocrystallized ligand

6	6W63	Gln189/O	2.28	Asn142, Glu166, Leu141, His41	−7.1	−8.3

	6XHM	NH/Glu166	1.92	Met165, His41, Pro168, Ala191, r190	−6.9	−7.2
		Glu166/O	1.98			
		NH/Gln189	1.93			
		O/Gln189	2.91			
		His163/O	2.30			

	7D1M	His41/O	2.14	Met165, Asn142, Glu166	−7.8	−7.3
		NH/Cys145	2.86			
		Cys145/O	3.42			
		Ser144/O	2.39			
		NH/Gln189	2.00			

8	6W63	NH/Glu166	2.27	Met165, Met49, His41, Pro168	−6.9	−8.3
		Gln189/O	2.98			
		OH/Cys44	2.84			

	6XHM	His41/O	3.07	Met49	−6.6	−7.2
		Cys145/O	3.65			
		Cys145/O	2.39			
		His163/O	1.99			

	7D1M	His41/O	2.67	Met49	−7.3	−7.3
		Cys145/O	2.52			
		Ser144/O	2.29			
		Ser144/O	2.55			
		NH/Gln189	1.96			
		Gln189/O	2.75			
		Glu166/O	2.29			

17	6W63	NH/Glu166	2.41	Phe140, Asn142, Leu141, Met49, His41	−8.1	−8.3
		Gly143/O	2.90			
		His163/N	1.91			
		NH/Cys145	2.85			

	6XHM	His163/N	2.18	Asn142, Leu141, Met165, His41, Met49	−7.4	−7.2
		Cys145/O	2.77			
		NH/His164	1.89			
		Gly143/O	2.03			

	7D1M	His41/O	2.78	Met49	−7.8	−7.3
		His163/N	2.49			
		NH/Cys145	3.71			
		Cys145/O	3.72			
		NH/Asn142	2.44			
		NH/Gln189	2.29			

**Table 7:** MM/GBSA ΔG binding scores of cocrystallized ligands and top three docked ligands.

PDB code	Ligand number	MM/GBSA ΔG bind ± SD* (kcal/mol)
6XHM	Cocrystallized ligand V2M	−84.6113 ± 5.11
	6	−58.5536 ± 4.86
	8	−52.0877 ± 7.19
	17	−76.4401 ± 5.52
7D1M	Cocrystallized ligand K36	−62.2082 ± 10.22
	6	−52.3283 ± 15.70
	8	−58.3482 ± 3.93
	17	−67.8806 ± 5.09
6W63	Cocrystallized ligand X77	−73.9525 ± 6.44
	6	−43.2766 ± 6.10
	8	−55.4145 ± 12.81
	17	−62.5424 ± 6.25

* SD, standard deviation

## Footnotes

1 World meter coronavirus [online]. https://www.worldometers.info/coronavirus/ [accessed 14 March 2021].

2 World Health Organization (2020). Coronavirus disease (COVID-19): Vaccines [online]. https://www.who.int/news-room/q-a-detail/coronavirus-disease-(covid-19)-vaccines [accessed 14 January 2021]

3 RCSB PDB. Protein Data bank [online]. https://www.rcsb.org/ [accessed 14 January 2021]

4 Molinspiration chemoinformatics [online]. https://www.molinspiration.com/ [accessed 05 February 2021]

5 BIOVIA, Dassault Systèmes, Discovery Studio Visualizer, 17.2.0.16349, San Diego: Dassault Systèmes, 2016.

6 Schrödinger Release 2020-4: Desmond Molecular Dynamics System, D. E. Shaw Research, New York, NY, 2020. Maestro-Desmond Interoperability Tools, Schrödinger, New York, NY, 2020.

7 Schrödinger Release 2020-4: Maestro, Schrödinger, LLC, New York, NY, 2020.

8 The PyMOL Molecular Graphics System, Version 2.4.1 Schrödinger, LLC
